# Epistemic deferral, gnoseology, and intellectual ethics

**DOI:** 10.1007/s44204-025-00287-7

**Published:** 2025-05-24

**Authors:** Jesper Kallestrup, Edoardo Cavasin

**Affiliations:** University of Aberdeen, Aberdeen, AB24 3 FX Scotland, UK

**Keywords:** Sosa, Virtue epistemology, Epistemic deferral, Gnoseology, Intellectual ethics

## Abstract

Sosa (2021) has recently presented a dichotomy between, as he puts it, the humanistic domain, in which we seek first-hand understanding of why-questions of value and normativity through our own intuitive insight, and the practical domain in which we defer to epistemic authorities for information that correctly answers utilitarian questions. However, this neat picture is too simplistic. To use Sosa’s terminology, whereas gnoseology also applies in the humanistic domain, intellectual ethics is very much equally about the practical domain. Moreover, the distinction between first- and second-hand knowledge does not coincide with any such distinction between domains of inquiry. In this paper, we first develop a virtue-theoretic account of epistemic deferral, which displays Sosa’s (2021) triple-*A* and triple-*S* structures, and reflects his different levels of telic assessment. We then show how this account not only pertains to both domains, but also explains why there is no real tension between conducting our epistemic inquiries autonomously and deferring to the epistemic authorities for knowledge that we are incapable of acquiring ourselves.

## Introductory remarks

In a series of hugely influential works Sosa (2007, 2009, 2011, 2015), Sosa has developed and defended a characteristically elegant *virtue epistemology* which is telic by way of explaining knowledge and cognate phenomena by appeal to virtues that aim at epistemic goods. The core idea is that knowledge is a cognitive achievement, which is an attempt (or a performance) that is successful through the agent’s skill when in suitable shape and situation. This view holds much explanatory promise. In particular, Gettier cases are ones in which the belief is true because of luck rather than skill. Sosa’s most recent iteration (Sosa, 2021) adds a new layer to earlier accounts. *Animal knowledge* is apt belief, where a belief is apt when accurate (i.e. true) because adroit (i.e. competent). That’s the *triple-A* structure, but the competence in question must also display a *triple-S* structure, comprising innermost skill, physical shape, and surrounding situation. Furthermore, *reflective knowledge* is fully apt belief, i.e., apt belief aptly acknowledged. It involves a second-order perspective of consciously endorsing cognitive performances on the first order. So, where the aim is accuracy on the first order, the aim is aptness on the second order. Finally, the newcomer *secure knowledge* is knowledge in the absence of nearby worlds in which the triple-*S* competence is lost while retaining the ability to make a judgment as to whether the target proposition is true.

Here’s the plan for the paper: Sect. 2 expounds Sosa’s telic virtue epistemology, especially as it pertains to knowledge. In (Sosa, 2021), Sosa also deploys this general virtue-theoretic framework to suspension of judgment, but (interestingly enough) not to epistemic deferral, even though this important cognitive phenomenon is treated in the earlier parts of this work. We shall seek to fill this lacuna by developing such an account in Sect. 3. We then proceed in Sect. 4 to argue that what Sosa (op. cit.) calls *gnoseology*, or the theory of knowledge, also applies, at least to a significant extent, in the *humanistic domain*, including philosophy and the arts, while what he calls *intellectual ethics,* with its focus on questions of value and normativity, is very much about the *practical domain* too, which includes medicine, finance, law, and science. In particular, Sosa (op. cit.) claims that understanding why, which he equates with intuitive insight, has a special value and standing in the humanities. In contrast with the questions asked in this domain, whose answers require *first-hand intellectual engagement* with matters of human flourishing, the questions asked in the practical domain can be properly settled through sheer deference to those in the know. For the latter, *utilitarian questions* merely call for information about factual matters. However, we argue from illustrative cases that both first-hand knowledge by rational insight, and second-hand knowledge by deference, are very much in play in both domains. In Sect. 5, we apply our account of *apt deferral* to these domains of discourse, showing that it suits both equally well. As the account combines competences of the deferring part with competences of the part deferred to, it overcomes the asymmetry Sosa alleges between such first- and second-person knowledge, as well as between rationally responding to disagreement with the epistemic authorities in those domains. And our account helps resolve a perceived tension between conducting our epistemic inquiries autonomously and deferring to such authorities for knowledge and understanding. Finally, Sect. 6 contains some concluding remarks.

## Sosa’s telic virtue epistemology

All virtue epistemologists assign a central role to epistemic (or intellectual) virtues in theorising about knowledge, or our epistemic lives more broadly. They disagree over how to characterise those virtues. *Virtue responsibilists*, e.g., Zagzebski (1996, 2000), understand virtues to include refined character traits, characteristic of responsible inquirers, such as conscientiousness, open-mindedness, perseverance, or humility. *Virtue reliabilists*, among which Sosa (2007, 2009, 2011, 2015, 2021) is arguably the most prominent, understand virtues to include stable and reliable cognitive faculties, such as perception, reasoning, introspection, or memory. What is distinctive of Sosa’s virtue epistemology is its *telic* (or teleological) aspect, which means that the virtues to which he appeals aim at epistemic goods. On his view, epistemic normativity is telic. Basically, attempts (or performances) attain the end to which they are directed when they succeed *through* (exercising or manifesting) competence. To use Sosa’s familiar terminology, an *achievement* is an *apt attempt*, and an attempt is apt when accurate *because* adroit. That’s the triple*-A* structure of achievements. In the cognitive domain, *animal knowledge*, which is the first grade of knowledge, is apt belief, where a belief is apt when its truth (accuracy) is owed to competence (adroitness).^1^ Such knowledge is thus a cognitive achievement. Bad luck can prevent a competent belief from being true, hence from being knowledge. But as Gettier (1963) taught us, even a belief that is both true and competent may fall short of knowledge. In such cases, as Zagzebski (1994) noted, the bad luck is cancelled out by good luck. To avoid knowledge-undermining luck, the truth of the belief must be down to competence. This virtue-theoretic constraint accounts for Gettier cases involving *intervening* luck, such as the fake sheep case,^2^ but not for cases of *environmental* luck, such as the fake barn case.^3^ However, that may well be a feature rather than a bug of his view. After all, empirical evidence from the X-phi literature suggests that folk judgements support the claim that the fake barn case is one of knowledge.^4^ This, however, remains a crucial point for considering the merits of the virtue-theoretic framework and, in the following, we will consider the explanatory power of Sosa’s account.

To see why Sosa’s account makes different predictions about cases of these different types of luck, we need to review his notion of a competence. Sosa (2009, 2015, 2021) adds to his triple-*A* analysis of knowledge a triple*-S* analysis of competence, where a complete competence combines skill, shape, and situation. More precisely, he distinguishes between an innermost (or constitutional) *S*-competence, which is the core skill seated in the agent; an inner *SS*-competence, which is the combination of skill and shape; and a complete *SSS*-competence, which is the conjunction of skill, shape, and situation. While skills are fairly stable, by way of supervening on intrinsic physical features of agents, shapes and situations are more variable. As an example, if the complete competence is being a competent driver of a vehicle on a certain occasion, then the skill is the basic driving ability, the shape is being awake, sober, alert, etc., and the situation is being seated at the wheel, in a reliable vehicle, on a dry road, etc. The connection between the triple-*A* analysis of an attempt and the triple-*S* analysis of a competence is the following: an attempt is apt when its success manifests competence, which happens just in case the skill *causally produces* the success in combination with an appropriate shape and situation.^5^ The skill of the competence is determined as the causal basis for a success-response of an object when subjected to a stimulus in certain shape and situation combinations. Since a *complete* competence is necessarily a competence to reliably succeed when trying for some outcome, such that if one tried, one would very likely succeed, no such complete competence can bring about that outcome. Only the skill can do that when the shape and situation are conducive to the outcome. Now, in the fake sheep case, the truth of the belief is not causally produced by the agent’s skill, i.e. their core ability of visually detecting sheep, because the situation, i.e. the conditions for observation, is abnormal in that what looks exactly like a sheep is in fact a cleverly disguised dog. But in the fake barn case, the situation is in actual fact favourable, and so the skill is causally responsible for getting it right. For when it comes to visual perception, the relevant conditions are *local*, and a real barn is in clear view, not blocked or covered by intervening lookalikes. The fact that the countryside is populated with facsimiles means Barney is lucky to manifest a triple-*S* competence, because the situation is modally unsafe, but that’s irrelevant vis-à-vis the conditions for seeing the barn in question.^6^ Aptness merely requires that such competence be manifest, irrespective of whether its possession is modally safe.

But what then explains the intuition, as reported by many epistemologists, that the environmental luck in the fake barn case is as incompatible with knowledge as the intervening luck is in the fake sheep case^7^? At this juncture, Sosa used to appeal to *reflective knowledge*, which, as the second grade of knowledge, is apt belief aptly guided.^8^ On this meta-level, one attains *full aptness* when one succeeds at aiming for aptness, which is more demanding than aiming for truth, because one manifests not only a first-order competence for hitting the truth, but also a second-order competence of being attuned to borderline conditions for exercising the former competence, i.e., a sensitivity to whether the selected situation is conducive to getting it right (so as to avoid recklessness or even negligence). Thus, in the fake barn case, Barney seems insensitive to which situation should be chosen for correct belief formation, because the real and fake barns are visually indiscernible from a distance. He lacks the meta-competence required for reflective knowledge. For that reason, Sosa’s old view was that while the fake sheep case and the fake barn case differ in that only the latter exemplifies animal knowledge, they are also alike in that neither exemplifies reflective knowledge.

However, in (2021: 170–186) Sosa claims that what Barney lacks is not reflective knowledge, for such knowledge can be based on *default assumptions*. All Barney needs is *apt awareness* that his belief will be, not just true, but also apt, and he is default entitled to assume that if the object perceptually appears to him to be a barn, then that is what it is.^9^ Or more generally in his telic framework (2021: 159), “agents can assume by default the satisfaction of background conditions”. Of course, in fake barn county, Barney could easily have been looking at a barn façade, and so, as he might easily have been mistaken, the competence provided to him by the real barn is not secure. It is retained unsafely, through luck. So, what he lacks is rather *secure knowledge* (full well), which an agent has just in case there are no nearby worlds in which they lose their triple*-S* competence while retaining the ability to make a judgment about whether *p*. Secure knowledge ranks above reflective knowledge in the *metaphysical hierarchy* of increasing attributability to the agent and decreasing agent-external luck (2021: 185–186), because once default assumptions are on board, one can reflectively know in some situations without one’s competence being securely in place.

## A telic virtue epistemology of deference

The basic category in Sosa’s (2021) taxonomy of telic normativity is that of *attempts*, which are equated with taking means that are aimed at a targeted objective. In the cognitive sphere, attempts are primarily judgments and (judgmental) beliefs, but Sosa (2021: chap. 3–5) also develops a novel account of the *suspension of judgment*, understood as an intentional forbearance from judging. However, even though he discusses the belief-forming process of *deference* in (2021: chap. 1), no telic virtue-theoretical account of this important notion is offered. This section seeks to do just that. Some preliminary remarks are in order.

First, by ‘deference’ we have in mind *epistemic* deference, where one defers to an *epistemic authority* for knowledge. A special case is semantic deference, where one exploits a division of linguistic labour by deferring to a linguistic authority to knowledgeably fix the extension (or intension) of some term or concept.^10^ We have a broader kind of epistemic deference in mind, which a division (or distribution) of cognitive labour *simpliciter* allows for. And since we shall take such division to comprise only human agents, epistemic authorities are similarly restricted to include only human agents.^11^

Secondly, we define ‘epistemic authority’ in terms of *epistemic superiority*, as follows:

(EA)*A* is an epistemic authority for *B* regarding subject matter *s* if and only if *A* is epistemically superior to *B* regarding *s*.^12^

And we shall understand such superiority in terms of having better and/or more evidential reasons, and being skilful at evaluating them, as regards *s*, where such reasons are *epistemic* by way of being truth conducive.^13^ Furthermore, for someone to be an authority, they must have sufficient reasons and skills to acquire a substantial amount of knowledge on the subject matter. While what counts as a substantial amount will vary across different subject matters, such *minimum threshold* for epistemic authority is needed to rule out the possibility that, say, one toddler counts as an authority in virtue of knowing next to nothing about some topic relative to an even younger toddler who literally knows nothing about that topic.

Thirdly, for convenience, we shall coin some new terms by stipulating the following:

(*S*)A deferral regarding subject matter *s* is a two-place relation between a deferrer and a deferee such that the deferrer defers in respect of *s* to the epistemically superior deferee.

Putting (EA) and (*S*) together, deferral thus always involves epistemic authority.^14^ Importantly, while deferees are relatively more reliable (with respect to *s*) than deferrers, they are not infallible, and so they may stand corrected. Alternatively, ‘epistemic authority’ can be construed *socially*, in terms of occupying professional roles, possessing official credentials, etc., but the problem with this proposal is the contingency of such conventional marks of esteem and acclaim, which could possibly render someone an authority in name only, thus preventing the deferential belief from constituting knowledge. We shall henceforth reserve the term ‘expert’ for a socially (rather than epistemically) defined epistemic authority.^15^

Fourthly, to acquire deferential knowledge, not only must the deferrer’s belief be *based on the fact* that the deferee holds that *same* belief, the deferee must also hold that belief *because of* their epistemic authority on the relevant subject matter. This last constraint is necessary to avoid cases in which a deferee holds a belief as a result of, say, guesswork, (intervening) luck, or bias.^16^ Imagine a leading historian of science who holds the true belief that Einstein is the author of “On the Electrodynamics of Moving Bodies”, but for the reason that Einstein was the first famous physicist that came to their mind. Because the belief is disconnected from their epistemic authority, a layperson deferring to that expert historian would fall short of deferential knowledge.

Fifthly, the deferrer must have a reasonably good grasp of who the deferee is, sufficient to distinguish the actual deferee from other possible deferees who all are epistemic authorities on the relevant subject matter. This requirement is called for to avoid cases in which deferential knowledge is jeopardised by deferrers confusing different deferees. Thus, suppose a layperson defers to epistemic authority *A*, ascribing her the true belief that *p*, although the layperson in fact confuses *A* with a different epistemic authority *B*. Because the layperson is mistaken about whom they defer to, such a case would not allow for the acquisition of deferential knowledge, even if indeed *A* does hold the belief that *p*.

With those observations in mind, let us turn to the question of how to build an account of epistemic deferral through the lens of Sosa’s virtue epistemology.^17^ Any example of a telic triple-*A* normativity should involve his five main ideas of attempt, success, competence, aptness, and achievement. And it should at least distinguish between first-order attempts at accuracy, which amount to achievements when apt, i.e., when accurate because adroit; and second-order attempts at aptness, which amount to achievements when fully apt, i.e., when apt through meta-competence. So, what would it take for a deferral to be *successful*? At a minimum, it should involve a deferee providing a true answer *p* (possibly, but not necessarily, through communicative exchange) to the question of the deferrer’s query. But, as noted earlier, we would not want the deferee to do so through (intervening) luck, as that would prevent any deferential belief from constituting knowledge, and so a deferral should be considered successful only if the deferee furnishes *p* through their relevant competence.^18^ And because deferees are epistemically superior, when *p* manifests their competence, the deferee would offer up more and/or better reasons than any reasons already possessed by the deferrer, or a more reliable answer to whether *p* based on them.^19^ Moreover, a deferral falls short if no true belief that *p* is actually formed as a result of deferring, or if, as mentioned above, a true belief is formed but the deferrer is labouring under a misapprehension about whom the deferee is. So, a deferral is successful just in case the deferrer forms a true belief that *p*, which answers their query, on the basis of the fact that the deferee, whom the deferrer correctly identifies, states that *p* because of their epistemically superior competence.

What then would make a deferral also *apt*, i.e., accurate because adroit? This question is about what competence of the deferrer would need to be exercised such that the success of the deferral, as per above, is because of it? The answer is: being *competent at identifying a relevant deferee*, i.e., an epistemic authority on the subject matter in question who can reliably deliver a true answer *p* to the deferrer’s query. Assuming, as is typically the case, that the epistemic authority is also an expert, such competence will normally manifest in knowing about pertinent professional roles or recognised degrees, sufficient to reliably identify such authority as and when needed. This proposal is attractive, because it explains how deferrers are able to identify the deferees to whom they must turn to acquire *p*.

Consider this version of the *expert identification problem*: if deferrers do not have the epistemic authority (on subject matter *s*), they are not able to reliably identify the right deferees, but if deferrers did have such authority, they would not need to identify any.^20^ On our view, the first horn is false. Deferrers are typically able to identify the right deferees by means of formal credentials, relying on those as a proxy for authority. So, the advice would be to only consult *certified, licenced,* or *accredited* authorities, i.e., what we are calling ‘the experts’, as these labels are indicators (though not guarantors) of reliability and trustworthiness. They are, in turn, underwritten by institutional bodies, which are a kind of *meta-experts* who have given their seal of approval to ensure that laypeople will be advised and treated in a fully qualified, committed, and accountable manner. While epistemic authorities are typically approved to advise or inform in a professional capacity, not all and only such authorities are experts, flagged up as reliable sources of information in this fashion; that is why we opted for an epistemic characterisation of an epistemic authority in (EA). Still, in a *normally functioning* epistemic community, where the cognitive labour is adequately distributed, and where such authorities are systematically organised, institutionally recognised, and conventionally flagged up, most experts will be epistemic authorities (and many, but not most, epistemic authorities will be experts^21^), and so deferrers will (often enough) be competent at reliably and effectively navigating their way through the community to the right deferees. In Sosa’s terminology, in such a *good case*, their deferral is thus apt, because successful through competence.^22^ Or more precisely, the deferral is apt because the innermost skill causally produces the success in combination with an appropriate shape and situation, where the important point for our purposes is that in the good case the surrounding situation is conducive to success.

There are also tools that deferrers can use to identify an epistemic authority who does not have the social markers that characterise experts. Nguyen’s (2018) *litmus test* is a case in point. The idea behind this test is that in many cases subjects can attribute epistemic authority when they can see the success of someone involved in a certain enterprise. Take the case of subject *A* for whom there are no social markers of epistemic authority in aeronautical engineering*. B* then discovers that *A* managed to successfully build a fully functioning airplane. Even though *B* knows nothing about the subject matter, *B* is able to aptly identify *A* as an epistemic authority, relative to *B*, in aeronautical engineering. This procedure, however, is still quite risky for a layperson who knows next to nothing about a particular field, since they can easily be misled. That is why, at least in what we described as a good case, the recommended strategy is still to defer to the experts, as they will most likely also be epistemic authorities.

So much for aptness of deferral. The next level of telic normativity is full aptness. So, what would it take for a deferral to be not just apt but fully apt? Put differently, what additional conditions would need to be met for a deferrer to defer in a reflective, and not merely animal, way? To fix ideas, contrast the good case with a *bad case* in which the epistemic community is *functioning abnormally*, but where this is *undetectable* to the deferrer. In particular, most of the experts in the bad case actually lack the epistemic superiority that is definitive of epistemic authority. However, no tell-tale signs would reveal to the deferrer which case obtains. Suppose further that Audrey, by exercising her first-order competence, has managed to identify one of the rare experts in the bad case who genuinely is an epidemic authority, and who does provide the true answer *p* that she needs to further her epistemic pursuits. Audrey is an ordinary layperson who has no clue that she is in a bad case. Thus, we have in effect a social counterpart of the fake barn case from Sect. 2.^23^ Audrey’s deferral is clearly apt, but is the aptness also aptly guided, i.e., does the aptness of her deferential belief manifest her meta-competence to attain aptness through (first-order) competence? Audrey would seem to lack such second-order competence of being attuned to borderline conditions for exercising her competence. And if she cannot discriminate between real and fake experts, she is insensitive to which situation should be selected for deferral. The *environmental luck* in the bad case thus suggests that Audrey’s deferral is not fully apt, or reflective; or so Sosa would have it in earlier work Sosa (2007, 2009, 2011, 2015).

However, Sosa’s (2021) view makes a different prediction about the bad case. All Audrey needs for reflective deferral is *apt awareness* that her deferral is apt. And once we add *default entitlements* to the mix, nothing could deny her such awareness. For just as Barney from Sect. 2 is entitled to assume that something is a barn if it looks like one, Audrey is entitled to assume that someone is an epistemic authority if, basically, they look like one (i.e., display all the socially recognised hallmarks of expertise). And such default assumptions can serve as bases for their respective perceptual and deferential beliefs, thus constituting knowledge if true and undefeated. Thus, Barney and Audrey can know that the barn and expert, respectively, are genuine.^24^

What Audrey’s deferral lacks is rather *security*, which, remember, sits above even reflective knowledge in the metaphysical hierarchy, ordered by the level to which the success is credible to the agent, rather than being down to luck. That is to say, while her deferral should be deemed both apt and fully apt, by the lights of Sosa’s (2021), it is not secure (full well), because there are nearby worlds in which her competence is lost while she would retain the ability to make a judgment as to whether an expert is likely to provide her with the true answer *p* that she needs to satisfy her epistemic needs. After all, in the bad case, Audrey is lucky to form a true deferential belief, because she could easily have formed a false such belief, had she approached one of the many indistinguishable experts who have no epistemic authority. Given how easily she might have been deceived, the competence provided to her by the genuine expert is insecure. It is retained unsafely, through luck. More precisely, it is the situation aspect of the triple-*S* competence that could so easily be lost^25^; not the skill or the shape, both of which we assume are fixed regardless of which case obtains. For security requires (op. cit., 171–174) that the competence be retained safely, which involves the safety of the “ostensible background conditions”. But in the case of Audrey, the competence is retained through luck, as she is lucky that those conditions (which basically constitute the situation) are conducive to forming a true deferential belief.

That completes our telic virtue-theoretic account of deferral to epistemic authorities, composing the distinct but ordered levels of animal, reflective, and secure deferral. In Sect. 4 we turn to Sosa’s (2021: chap. 1) distinction between the humanistic domain and the practical domain. Pace Sosa (2021: chap. 2), our argument is that what he calls *gnoseology*, which includes deferential knowledge, and *intellectual ethics*, which concerns first-hand understanding, very much apply in both domains. With that in mind, we revisit our account of apt deferral in Sect. 5 to show why there need be no tension in any domain between conducting epistemic inquiries autonomously and deferring to the epistemic authorities.

## Gnoseology and intellectual ethics

In (2021: chap. 2), Sosa draws a distinction between two branches of epistemology. First off, there is *gnoseology*, which is equated with a theory of knowledge. In particular, his telic virtue epistemology is an account in gnoseology. This is basically the project of teasing out individually necessary and jointly sufficient conditions for knowledge that *p*, or higher grades of such propositional knowledge, including reflective and secure knowledge. All reasons are here considered purely epistemic, sealed against extraneous practical incursion, and all assessments of beliefs (or other cognitive attempts) are wholly telic. Telic gnoseological assessments, i.e., assessments of a thinker’s cognitive telic performance, such as attempts at success, aptness, or full aptness, are unaffected by practical considerations.

Secondly, there is *intellectual ethics*, which involves very different evaluations of one’s intellectual agency. This is about what inquiry should be pursued, what questions to ask, and how to go about answering them, which may well depend on practical reasons. Here the aim is *understanding why* it is so that *p*, which in turn is correlated with knowing why it is so that *p*. Understanding why is *gradable*. My understanding of why some cars overheat in traffic is to a lesser degree than that of a mechanical engineer. Furthermore, one understands something well enough when one really understands it outright. While I have some understanding of the cooling system in a car, a mechanical engineer understands it outright.

Sosa also draws a distinction between the *humanistic domain*, e.g., philosophy or the arts, and the *practical domain*, e.g., medicine, law, finance, or science. In particular, understanding why has a special standing in the humanities. In contrast with the questions asked in this domain, whose answers require *first-hand* assessment and *autonomous* judgment of *normative* or *value*-laden matters of human flourishing, the questions asked in the practical domain can be properly settled through *sheer deference* to the right epistemic authorities. For the latter, *utilitarian* questions merely call for information about factual matters. By way of example, contrast questions about, say, the nature of morality or the appreciation of works of art, with questions about vaccine efficacy or inheritance tax.

Now, a tempting conclusion to draw is that gnoseology only pertains to the practical domain, whereas intellectual ethics applies exclusively to the humanistic domain. After all, gnoseological normativity is telic, which is “quite orthogonal” to the normativity of intellectual ethics (2021: 41). For the gnoseological aim is knowledge, which settles the questions of utility asked in the practical domain, whereas the aim of intellectual ethics is understanding why, which results from first-hand engagement with the questions of value or normativity posed in the humanistic domain. However, that cannot be right. To see why, let us delve deeper into these complexities, beginning with Sosa’s (2021: 6–7) take on understanding (well enough) why:

(UW)“You understand well enough why *p* if and only if you know well enough why *p*, which requires knowing, for some fact, that *p* because of that fact”.

Two observations about (UW). First, Sosa does not explain the ‘because’ relation in (UW), but we take it to be *causal explanatory*. Second, the word ‘requires’ merely suggests a necessary condition. What else is needed for enhanced understanding is more propositional knowledge, properly integrated. Let us say understanding why is *deeper*, the longer one knows a causal chain to (possibly) be: one knows that *p* because of *q*, and one knows that *q* because of r, etc. And let us say understanding why is *broader*, the more branches one knows a causal chain to (possibly) have: one knows that *p* because of *q1*, and one knows that *p* because of *q2*, etc., where *q1* and *q2* may be jointly sufficient causes of the same effect, or individually sufficient causes of different effects. Thus, all I know is that cars overheat because of inadequate coolant, but a mechanical engineer also knows that low coolant levels are because of either a failing head gasket, coolant hose leaks, or gradual coolant consumption by the engine. So, the engineer’s understanding why exceeds mine both in terms of depth and breadth.

Now, a pressing question is: why should not (UW) apply equally in our two domains of gnoseology and intellectual ethics? After all, (UW) has it that understanding (well enough) why *reduces* to (enough) propositional knowledge. So far, the only differences between intellectual ethics and gnoseology that Sosa points to concern the alleged connections between states of knowledge and their contents. In the former domain, one’s knowledge states must be connected along either or both of the dimensions we described, and their contents must pertain to causal explanation. Sosa does not place any constraints on connections between knowledge states or the nature of their contents in the latter domain, and so it would seem that (UW) *could* apply equally well in both domains. In fact, given that the need to understand why is no less evident in science (or in medicine, finance, or law) than in philosophy, the arts, and other branches of the humanities, it seems that (UW) *should* apply (more or less) to the same extent in both domains.^26^

On Sosa’s view (2021: 7–11), the key epistemological difference between the domains of the practical and the humanities is rather due to an *asymmetry* between how one initially *acquires* and subsequently *sustains* understanding why. In the practical domain, where one merely seeks answers to questions of utility, there are supposedly no constraints on how answers to those factual questions are first attained and then maintained. In particular, *mere deference* to epistemic authorities would do just as well as autonomous inquiry, relying solely on one’s own competences. But in the humanistic domain, a *direct rational appreciation* is called for. Humanistic inquiry is mostly *individual*, with *no sheer deference* to others. For instance, to understand why an artwork is aesthetically successful, one needs insight into the rational grounds for that success, which requires appreciating those reasons through *first-hand* experience. A critic may convey to the novice that certain features are what make the artwork successful, and so correct aesthetic judgment from first-hand competence may thus depend on the “conduit to reasons provided by testimony” (2021: 12).^27^ In that way, the critic provides *epistemic guidance* that enables the novice to understand why the work is successful.

Now, Sosa is of course right that if such understanding why relies on aesthetic *experience*, which in turn involves perceptual experience, it is hard to see how understanding why could possibly be imparted by a critic. But that is a special case of phenomenal knowledge, e.g., knowing what it is like to see red, which arguably requires undergoing distinctive experiences of phenomenal characters. Most understanding why in the humanities is not like that. Let us take a case of artistic expertise discussed by Fricker (2006). I want to know what an especially good example of an early nineteenth-century English transfer-printed cup and saucer is. Here, as a layperson on the topic of English pottery, my phenomenal experience is hardly of any help. I need the guidance and testimony of an expert (with epistemic authority) in order to understand, and therefore to know, why a particular cup is not only the result of a particularly skilful application of the transfer-print technique, but also a work that represents the general aesthetic ethos and taste of early nineteenth-century English ceramic art. My own insight and taste are still useful, but it will just point towards me finding the cup beautiful, given my contemporary aesthetic sensibility. Whereas deferring to an expert, as noted by Fricker (2006: 238), can have the effect of making me a bit of an expert myself.

Or take instead a moral case, also discussed by Sosa (2021: 9). Suppose a teenager defers to his parents for moral advice, as he lacks first-hand appreciation of whether his action was morally bad, and hence what he should do next. The parents saw what happened, and they advise their son to apologise, which he does. The explanation of the harm caused by the action gives the son some understanding of why an apology is due, but such second-hand knowledge may fall short. His understanding is truncated if soon thereafter he repeats the same action, completely oblivious to the upset caused and the need to apologise. Understanding through first-hand, i.e., non-deferential, knowledge is salient for normative or value issues in the humanities generally. And such humanistic understanding is desirable, in part because understanding of values and normativity should guide rational agency, and in part for the satisfaction of our curiosity; or so Sosa (op. cit.) maintains.^28^

However, this *epistemic asymmetry* between our two domains is too simplistic. Firstly, while much knowledge in the practical domain is second-hand, “first-hand intuitive insight”, or autonomous reflection, is also very much required. Following Sosa (op. cit.), law is a paradigmatic discipline in this domain, so take one of the *Waiting and Parking* sections of the UK Highway Code. Suppose one knows the exact formulation of rule 251 that states, “It is especially dangerous to park on the road in fog. If it is unavoidable, leave your parking lights or sidelights on”. Still, if one is unable to apply this rule in concrete cases, such as that mist or haze could also trigger its application if they significantly impair visibility, then one does not really understand it outright. And that requires first-hand reflection, if not “intuitive insight”. Of course, one may also learn from others that this case illustrates 251, but if one cannot explain why, or is unable to apply it in other obvious cases, then one’s understanding is at least very limited. What is needed is not more second-hand knowledge, but individual grasp or unaided comprehension of what the rule says, how it works, and what it implies. This is necessary both in the case of a truth-directed inquiry (think of a police officer or a road traffic lawyer who must know and understand the Highway Code in detail), and in the case of a utilitarian inquiry (where ordinary citizens need some familiarity with what the Code says and implies for their own driving in order not to incur fines or get penalty points).

Secondly, while thinking critically for oneself is certainly characteristic of the humanistic domain, there is an equally important role for deference to play. As, following Sosa (op. cit.), ethics is a paradigmatic discipline in this domain, let us consider Rawls’ (1971) *method of reflective equilibrium*. Philosophers make considered judgments about particular cases of justice, be they actual or merely possible. Maybe these judgements are based on intuitions, as a kind of intellectual seeming, elicited by such cases, but to say they are considered means they are made consistently and confidently. Philosophers also hold theoretical beliefs about the principles of justice that they take to govern or explicate those cases. The method of reflective equilibrium is then the deliberative process of working back and forth between such judgements and beliefs, so as to reach an acceptable coherence amongst them all. The point is now that one could learn much about this method from expert philosophers. For instance, one could only acquire second-hand knowledge of how Rawls (op. cit.) used it to arrive at his own *difference principle* and *equal liberty principle*. True, just as with the rules of the Highway Code, one would need to think carefully on one’s own feet if one were to deploy Rawls’ method to new cases, but that presupposes a significant amount of such prior knowledge stemming from others. Even in the more mundane case of an inexperienced parent trying to school their child, the best course of action is often learnt from others. For instance, when teaching principles of good study skills, such a parent may want to consult an educational expert for advice on how best to approach this pedagogical task.^29^

The upshot of the foregoing is that, pace Sosa (op. cit.), there is no obvious asymmetry between the practical and the humanistic domains in how understanding and knowledge are acquired and sustained. Both first- and second-hand knowledge are indispensable if one is to fully understand key features of both domains. Some knowledge is arguably more first-hand than second-hand, while other knowledge is more second-hand than first-hand, but that distinction does not neatly align with distinct domains of discourse. And that will not be any less true in the future as research conducted in these fields, including philosophy, is becoming more and more *interdisciplinary*. Not only are disciplines often viewed from the perspective of others, so that knowledge can intersect and insights transfer across them, perspectives and approaches from different disciplines are also increasingly integrated through collaborative interaction. Such synthesis can occur at the methodological level or in terms of theoretical frameworks. The shared goal of interdisciplinary research is often to achieve new knowledge by unifying a mix of existing first- and second-hand knowledge.

## Epistemic autonomy versus epistemic authority

With the foregoing criticism of Sosa’s epistemic asymmetry between the practical and the humanistic domains in mind, let us now revisit our account of apt deferral from Sect. 3. We focused on deferral to an epistemic authority on a subject matter *s*, which was defined in terms of epistemic superiority, as per (EA). So, by (*S*), the deferrer is epistemically inferior to the deferee, relative to *s*. The account also respected the different levels of telic assessment: success, aptness, full aptness, and secure aptness. More precisely, a deferral is successful when the deferrer forms a true belief that *p*, which answers their query, on the basis of the fact that a deferee, whom the deferrer correctly identifies, states that *p* through their epistemically superior competence. And a deferral is apt (or animal) when its success, thus understood, manifests a competence at identifying a relevant deferee on the subject matter *s*, where such competence consists in being able to discern a reliable source on *s* (who can provide the true answer *p*), typically on the basis of socially recognised hallmarks of their expertise, e.g., professional roles. Moreover, an apt deferral is fully apt (or reflective) when the deferrer is aptly aware that their deferral is apt, where such awareness is easily attained as the deferrer is default entitled to assume that someone is an epistemic authority if they appear in every way to be an expert. And finally, a fully apt deferral is secure if their triple-*S* competence would not easily be lost while they would retain the ability to make a judgment as to whether a relevant expert is likely to provide the true answer *p* to the deferrer’s query.

Now, let us make the independently plausible assumption that our practical and humanistic domains are both *epistemically good*, i.e., that these domains are normally functioning such that nobody is systematically deceived in the way Audrey was in Sect. 3.^30^ In fact, let us assume that someone is an expert (as defined in terms of social markers of esteem and acclaim) if and only if they are an epistemic authority (as defined in (EA) by epistemic superiority). That means a deferral is guaranteed to be secure if fully apt. We shall also assume that a deferral is fully apt if apt at all. Again, since default entitlements are easy to come by, and since we only ponder good cases where such epistemic standings are not defeated, we can safely ignore cases where apt deferrals are not also fully apt (or reflective). So, with those assumptions onboard, what holds of apt deferrals is also true of the other more demanding grades of deferral. We shall therefore restrict attention to apt deferrals.

What is striking is the contrast between our account and both of what Sosa (2021: chap. 1) calls *sheer deference* and *conduit-to-reason deference*. The former applies in the practical domain when utilitarian questions are properly answered with mere information provided by epistemic authorities. The latter applies in the humanistic domain when epistemic authorities provide epistemic reasons which are then adopted by rational insight as part of first-hand understanding why. Apt deference differs from sheer deference in that our account involves success through competences of not only the deferee, but very much also of the deferrer. Apt deference also differs from conduit-to-reason deference, because our account is not merely about the provision of authoritative reasons on the basis of which beliefs may then be formed by further rational insight, but rather it directly concerns the formation of beliefs in contents provided by the relevant deferee. Our examples in Sect. 4 from aesthetics, morality, and political philosophy are all cases in point.

What is more, our account of apt deference finds application in both of Sosa’s domains. By way of illustration, take again our examples from Sect. 4. When aptly deferring in the practical domain for knowledge of the Highway Code, the deferrer is drawing on their own competence at identifying a relevant expert who knows the true answer to the query. The deferrer uses conventional social markers to pick out a traffic police officer, or, if need be, a road traffic lawyer. But since the truth of the belief formed is very much down to the deferee, the deferrer is also relying on them to provide the correct answer through their epistemically superior competence such that the belief may amount to knowledge. Deferring in the practical domain thus involves exercising mixed competences on both parties. Similarly, when aptly deferring in the humanistic domain for knowledge, say, of the method of reflective equilibrium, one depends heavily on the competences of others to articulate and explain its content. Again, the right expertise will be identifiable via conventional social markers, such as university degrees. Crucially, what those deferees convey is the content of an answer to the query, rather than supporting reasons. That is not to say individual reflection and autonomous judgement are superfluous; thinking on one’s own feet is imperative for all understanding why, across different domains of discourse.

Let us finally turn to a perceived tension between epistemic deferral and first-hand knowledge. On the face of it, they seem to pull in opposite normative directions. For instance, Zagzebski’s (2012, 2013) *pre-emption view* says that when presented with an epistemic authority, we are rationally required to adopt their beliefs on the exclusive basis of their epistemically superior testimony.^31^ In contrast, Hazlett (2016) argues that non-deferential beliefs are socially valuable, not so much because they manifest our capacity for intellectual self-direction, but (in part) because they increase collective reliability. However, our account of apt deferral assigns, as we have seen, indispensable roles to both first- and second-hand knowledge. In cases where the epistemic authorities are also socially flagged up as reliable sources of information, a deferrer is able to consult them for answers to their epistemic inquiries. But such deference does not imply wholesale replacement of the deferrer’s own reasons with authoritative reasons. The alternative *total evidence view*, as in Jäger (2016), Dormandy (2018), and Lackey (2018), accommodates this point by saying that the deferrer’s belief should rationally be based on the total evidence that they possess, instead of being based solely on the belief or evidence of the deferee, i.e., no matter how forceful or plentiful the authoritative reasons are, they never replace or pre-empt the deferrer’s own reasons. Still, proponents of the total evidence view say that deferrers ought to assign the authoritative testimony of deferees *significant* evidential weight.^32^

Our account of apt deferral combines well with the total evidence view, for any apt deferral will involve a mix of reasons provided by the competences of both parties. Moreover, the account is neutral about *level splitting* in considering the evidence. If a deferrer wants to consider the evidence about the reliability of a deferee as second-order evidence,^33^ then the result of apt deferral will enter into the deferrer’s judgment as a factor that increases their level of confidence in the first-order evidence acquired from the deferee. After all, by identifying a suitable deferee for the query in question, the deferrer increases the probability of getting the right answer, and thus excludes any malfunction in their belief-forming process. If, instead, the deferrer considers all the evidence on the same epistemic level, then the result of apt deferral will still be part of their evidence basis. The fact that the deferee has been competently judged to be an expert would be one more piece of evidence in favour of aligning the final belief to that of the expert. In both cases, our account nicely explains the intuitive appeal of the total evidence view yielding the same result.

The total evidence view emphasises the importance of balancing all good evidence, regardless of whether its source is epistemically superior or inferior, so as to attain the highest degree of doxastic justification, but what happens when *disagreement* seems to be never ending? The humanities are rife with disagreement, even among epistemic peers, with no resolution in sight. Think of ancient-old debates in philosophy over free will, the value of knowledge, or principles of justice. But, as Sosa sees it (2021: 11–12), such disagreements can properly be discounted in the humanities. For disagreement is a special case of testimony, and once we aim for first-hand knowledge in pursuit of understanding, i.e., for knowledge through our own competences, we can safely ignore second-hand knowledge via deference. When faced with disagreement, say in philosophy, we should remain steadfast and resist pressure to revise for that reason. In fact, we should rarely be moved to assign much weight to what others say in this domain, no matter their standing; or so Sosa has it. That is not to say we should not engage in dialogue, or exchange views, or reconsider our own view as a result thereof, but that we should still aim to judge autonomously. Nor is that to say correct judgment from first-hand competence cannot be guided by reasons provided by expert testimony. What Sosa (op. cit.) has in mind here is again *sheer* deference to, or merely relying on, others for knowledge, as opposed to such “conduit to reasons” deference.

Sosa’s view on disagreement and deference, however, introduces a tension between understanding why and knowing why. As mentioned in Sect. 4, according to Sosa’s (UW), understanding why is reducible to knowing why. Moreover, the amount of knowledge why possessed is proportional to the depth and/or breadth of corresponding understanding why (2021: 7). The more propositional knowledge of the form ‘*p* because of *q*’ one has, the more enhanced is the resulting understanding along either or both of those dimensions. Such parallelism should obtain in both of Sosa’s domains, but where in the practical domain knowledge why can be the result of sheer deference at the expense of final understanding (2021: 4), in the humanistic domain the first-hand knowledge allows for a different and, arguably, superior kind of understanding enriched by rational insight. This, however, comes at a cost to the *explanatory power* of Sosa’s theory of understanding. For what could first-hand knowledge possibly bring to understanding that is not just more propositional knowledge? And how does this new element interact with the gradability of the two notions of understanding why and knowledge why? The formulation of understanding why in (UW), appealing as it is, cannot account for this added distinction between humanistic understanding of questions about value and normativity, and practical understanding of utilitarian matters. Moreover, discounting disagreement with experts in the humanistic domain adds a further tension between understanding why and knowledge why. If, as per (UW), the former is reducible to the latter, it should not be the case that what would arguably provide more propositional knowledge why—namely, deferring to those experts—is detrimental to gaining understanding why, which is the primary epistemic objective in the humanities.

Once we cease to sharply separate the humanistic and practical domains, however, the twin tensions between (UW) and its application to both domains, and between sheer deferral for utilitarian questions and steadfastness for value and normative issues, both disappear. The practical domain requires understanding why and knowledge why just as much as in the humanistic domain. A deep and broad understanding of the true answers to utilitarian questions is distinctively valuable, as is the knowledge acquired through deferral in the humanistic domain. To use one of Sosa’s examples (2021: 13), humanistic judgments are comparable to crossword solutions. To be told the answers to a crossword puzzle defeats the purpose, which is to get them right through first-hand reasoning, drawing on one’s own knowledge. That’s compatible with being coached by a cruciverbalist who can offer instructive tips and advice, e.g., heuristics such as: fill in the blanks first, or: confirm an answer by solving the entries that cross it. But, as we argued, the same is true in the practical domain. To be told the literal content of 251 of the Highway Code is to no avail if one fails to understand through first-hand reflection what this rule means for a range of concrete cases. A road traffic officer may offer some explanatory guidance, but ultimately one is going to have to grasp the rule by thinking through its implications and applicability to cases.

## Concluding remarks

We began with the observation that Sosa, despite discussing epistemic deferral in the first part of his (Sosa, 2021), never applies his influential virtue-theoretic framework to this important cognitive phenomenon. In the positive part of this paper, we proposed a telic virtue epistemology of such deferral, where the deferrer autonomously inquires in order to find a suitable deferee, i.e., a source that is epistemically superior to the deferrer on the subject matter in question, so as to satisfy their epistemic needs. Our account reflects Sosa’s distinct grades of telic assessment: successful deferral (where the deferral brings about a true belief largely through the deferee’s superior competence), apt deferral (where the success of the deferral is mostly due to the deferrer’s competence), fully apt deferral (where the deferrer is aptly aware, through default entitlements, that their deferral is apt), and secure deferral (where the deferrer’s competence would not easily be lost while retaining the ability to make a judgment as to whether a deferral is likely to be successful). We then observed that our account has the attractive feature of allowing for epistemic autonomy even in cases of apt deferral. This brought us to the next section, where we criticized Sosa’s distinction between utilitarian questions, which can be settled with sheer deferral, and humanistic questions, which need a deeper or broader understanding why. We argued, via illustrative cases, that even utilitarian questions call for understanding why beyond sheer deference, while in the humanistic domain, deference still plays a crucial role. More specifically, we showed how, by the lights of our telic virtue-theoretic account of deferral, the humanistic domain can allow for deferential knowledge without compromising the need for such understanding. Finally, we noted how, once we cease to drive a wedge between humanistic and utilitarian questions, our account of apt deferral not only combines well with the total evidence view of how to rationally respond to epistemic authority, it also resolves any perceived tension between epistemic autonomy and deferring to such authority.^34^,^35^

## Footnotes

1 In line with earlier work, Sosa (2021: 24–5) talks about alethic affirmations that *p*, which are attempts to get it right by affirming that *p*. Judgments are then attempts to get it right *aptly* by alethically affirming that *p*. He illustrates the difference by the quiz show contestant who aims to answer correctly by merely guessing, and the expert oncologist who aims to get things right through competence. He (op. cit. 144) does allow for cases of animal (“sub-credal”) knowledge without judgmental belief, such as non-accidental guessing.

2 Another promising feature of Sosa’s view is its ability to handle the Meno problem of why knowledge is valued more highly than true belief. Since being accurate because competent is more valuable than being both accurate and competent, but not accurate because competent, knowledge qua achievement has distinctive epistemic value. Its value corresponds to the credit or praise we deserve when forming apt beliefs, which exceed any credit or praise assigned on the basis of mere accuracy and competence. See Sosa (2007) and Kvanvig (2003).

3 The fake sheep case is originally due to Chisholm (1977), whereas Goldman (1976) credits the fake barn case to Carl Ginet. For more on intervening and environmental luck, see Pritchard (2015). Note that all knowledge-undermining luck is veritic, as it pertains to the truth of the belief. One may also be lucky to form the belief in question, say at the right time or place, but that implies at most that one is lucky to know on this occasion.

4 See, for example, Turri (2017, 2018).

5 One might distinguish between direct and indirect causal production. To illustrate, the goals Messi scores manifest his football abilities, but his salary constitutes no manifestation of those abilities. Only the goals are caused directly by his innermost skill when in the right shape and situation. His innermost skills merely play an indirect causal role in accounting for the astronomical pay Messi receives.

6 See also Kallestrup and Pritchard (2014).

7 This is the case for propositional knowledge. As far as practical knowledge is concerned (i.e., the kind of knowledge for which, contrary to propositional knowledge, the direction of fit goes from the intention of the subject to the external world (Anscombe 1963)), this intuition is less common. For an account of why practical knowledge is immune to environmental luck, see Carter and Pritchard (2015). However, as we are not concerned with practical knowledge, we shall henceforth use ‘knowledge’ to indicate propositional knowledge.

8 In earlier work, Sosa distinguished between reflective knowledge, which was about merely aptly noting or recognising that one’s belief is apt, and knowledge full well, which was a belief that is apt because guided by, or formed through, a second-order competence (say, to defend the belief against sceptical challenges). Basically, reflective knowledge was a belief that is both apt and fully apt, whereas knowledge full well was a belief that is apt because fully apt. In (2021: 55, 138–9, 147, 169, 186), Sosa talks about “reflective knowledge full well”, where the former is part of the latter, but for simplicity, we shall just refer to reflective knowledge.

9 Now, it may be that Barney’s belief is based on default entitlements, because he has a right to assume, indeed maybe he’s aware of that right, that things are as they perceptually appear to him, but it still does not follow that Barney’s belief is fully apt. A default entitlement is different from the kind of meta-competence Barney would need for his apt belief to also be aptly guided. We shall not pursue this line further, though see fn. 24.

10 Familiar from Putnam (1975) and Burge (1979).

11 There is an argument to be made for reliable instruments (e.g., thermometers), or indeed encyclopaedia, including Wikipedia, to be epistemic authorities, and for our competent use of them resulting in a kind of deferential knowledge. The chief reason for our restriction is that we understand epistemic authority in terms of the superior possession of evidence and evaluative skills. A more liberal construal of epistemic authority to include non-human resources is beyond the scope of this paper. We owe this point to an anonymous referee.

12 See also Goldman (2001), Zagzebski (2012), Wright (2016), Jäger (2016), Dormandy (2018), and Bokros (2021).

13 To say that epistemic superiors have more reasons is to be understood in terms of epistemic power or productivity. See also Goldman (1986) and Kelp (2023). One may suspect that our account implies that all standard cases of testimony turn out to be cases of deferral, in that they involve the transmission of knowledge, and so there must be an epistemic asymmetry. But even if we concede that a single whether-question could constitute a subject matter, there may be cases of testimony that are not cases of deferral. For epistemic superiority requires not just an asymmetry in the possession of evidential reasons, but also an asymmetry in skilfulness at processing them, and there may be cases of testimony where the testifier has more knowledge, because they have more/better reasons, but is less skilful at evaluating them than the testifiee, and so these would not count as cases of deferral in our sense. In any case, our positive view is not about testimony, at least in the narrow sense where a speaker makes an assertion aimed at a specific hearer. For instance, there are (also) cases of deferral without testimony. For instance, on this narrow construal, someone overhearing a testimonial exchange (or is otherwise eavesdropping) is not being testified to, but they could well form a deferential belief on that basis. Thanks to an anonymous referee for raising this point.

14 Worsnip et al. (2025) point to a divergence between certain normative ideals and the actual practice of deferral. For instance, the less skilful and knowledgeable you are, the more you ought to defer, but in reality, the less likely you then are to defer, in part because you are more prone to overestimating your own competence, and in part because the less competent you are at determining which experts to defer to. Likewise, you ought to defer less when there is disagreement amongst the experts, but you do not always suspend judgment when experts disagree, e.g., you may pick and choose, often depending on who shares your values and ideology.

15 Levy (2007), Harris (2023), and Brooks (2023) defend a social definition of ‘epistemic authority’.

16 Thanks to an anonymous referee.

17 The following account of apt deferral and deferential knowledge is based on the framework of Sosa’s telic virtue epistemology, but similar accounts could well fit into other competing virtue-theoretic frameworks, e.g., Greco’s (2010). Thanks to an anonymous referee.

18 For the answer *p* of the deferee to constitute an achievement, this competence of theirs would in turn need to display the triple-*S* structure of skill, shape, and situation. However, since our interest is in the act of deferral, we shall not delve deeper into the aptness of the deferee’s deliverances.

19 To require that the deferee also knows *p* is too strong. Lackey (2008) showed that one can acquire testimonial knowledge from a competent source that lacks knowledge, and the same is true of deferential knowledge.

20 See, for example, Goldman (2021).

21 Given our definition of ‘epistemic authority’ in terms of epistemic superiority, relations of such authority will be plentiful, whereas experts will be comparatively few. Thanks to an anonymous referee for this point.

22 Solving the expert identification problem still leaves the distinct problem of how a deferrer can avail themselves of authoritative reasons via deferral. Given that those reasons are better than their own, as they stem from a superior source, would they not need to be an authority themselves to form beliefs based on them, in which case deferral would be unnecessary? We return to this worry in Sect. 4.

23 See also Kallestrup and Pritchard (2012).

24 The objection from fn. 9 also applies to the case of Audrey. For her deferral to be fully apt, she needs to succeed through meta-competence, but basing a belief on a right to make certain assumptions about the world being the way it appears to be seems different from exercising any such competence.

25 As Sosa (2021: 170) puts it regarding a similar case, “she is not securely so located”.

26 It is certainly possible to reject Sosa’s distinction between the practical and humanitic domains, and to critique his account of first- and second-hand reasoning, without accepting our account of apt deferral. However, since Sosa argues for the distinction between those domains on the basis of the claim that in the practical domain sheer deference is allowed, if not encouraged, whereas features of the humanistic domain strongly disincentivise any such deferral, we take the two issues to be deeply intertwined. Since our account of apt deferral is not wedded to either of the two methods of inquiry that supposedly correspond to these distinct domains to the exclusion of the other, adopting it puts real pressure on Sosa’s distinction.

27 We return to Sosa’s notion of conduit-to-reason deference in Sect. 5.

28 Compare with Hills (2009), who argues that deferring to experts about moral matters is problematic, because moral beliefs formed on the basis of deference cannot achieve the kind of moral understanding that is required for acting with moral worth.

29 See also Fricker (2006).

30 Epistemically bad cases would only put more pressure on Sosa’s distinction between practical and humanistic domains. In contexts like the one Audrey finds herself in, the sheer deference that characterises practical domains would mostly fail to yield knowledge, thereby forcing her and other deferrers in a similar predicament to engage in first-order inquiries to avoid being systematically misled by fake experts. Such cases would de facto collapse any boundary between the two domains, creating a single context where deference only plays a minor role.

31 See also Keren (2014), and Constantin and Grundmann (2020).

32 Lackey (2018: 239) claims that “the testimony of experts should always be regarded as a piece of evidence to be weighed with the other relevant evidence we have on the matter”. And Worsnip et al., (2025: 4) define ‘deference to an expert’ in terms of coming “to believe the content of an expert’s testimony because one affords the fact that the expert testified to it considerable [epistemic] weight”.

33 See also Christensen (2010) and Feldman (2005).

34 We do not analyse or generate any datasets, because our work proceeds within a theoretical and mathematical approach. This research involves no humans or animals, and so no study-specific approval by any appropriate ethics committee is needed. There are no financial conflicts of interest either.

35 Many thanks to two anonymous referees for helpful comments and constructive suggestions on an earlier version of this paper. We are also grateful to the Arts and Humanities Research Council (Grant No. AH/W008424/1) for funding this research.
